# Development of a Specific Anaerobic Field Test for Aerobic Gymnastics

**DOI:** 10.1371/journal.pone.0123115

**Published:** 2015-04-13

**Authors:** Christiano Robles Rodrigues Alves, Marcello Tadeu Caetano Borelli, Vitor de Salles Paineli, Rafael de Almeida Azevedo, Claudia Cristine Gomes Borelli, Antônio Herbert Lancha Junior, Bruno Gualano, Guilherme Giannini Artioli

**Affiliations:** 1 School of Physical Education and Sport, University of São Paulo, São Paulo, São Paulo, Brazil; 2 Aerobic Gymnastics Department, Sociedade Esportiva Palmeiras, São Paulo, São Paulo, Brazil; Texas A&M University, UNITED STATES

## Abstract

The current investigation aimed to develop a valid specific field test to evaluate anaerobic physical performance in Aerobic Gymnastics athletes. We first designed the Specific Aerobic Gymnast Anaerobic Test (SAGAT), which included gymnastics-specific elements performed in maximal repeated sprint fashion, with a total duration of 80-90 s. In order to validate the SAGAT, three independent sub-studies were performed to evaluate the concurrent validity (Study I, n=8), the reliability (Study II, n=10) and the sensitivity (Study III, n=30) of the test in elite female athletes. In Study I, a positive correlation was shown between lower-body Wingate test and SAGAT performance (Mean power: p = 0.03, r = -0.69, CI: -0.94 to 0.03 and Peak power: p = 0.02, r = -0.72, CI: -0.95 to -0.04) and between upper-body Wingate test and SAGAT performance (Mean power: p = 0.03, r = -0.67, CI: -0.94 to 0.02 and Peak power: p = 0.03, r = -0.69, CI: -0.94 to 0.03). Additionally, plasma lactate was similarly increased in response to SAGAT (p = 0.002), lower-body Wingate Test (p = 0.021) and a simulated competition (p = 0.007). In Study II, no differences were found between the time to complete the SAGAT in repeated trials (p = 0.84; Cohen’s d effect size = 0.09; ICC = 0.97, CI: 0.89 to 0.99; MDC_95_ = 0.12 s). Finally, in Study III the time to complete the SAGAT was significantly lower during the competition cycle when compared to the period before the preparatory cycle (p < 0.001), showing an improvement in SAGAT performance after a specific Aerobic Gymnastics training period. Taken together, these data have demonstrated that SAGAT is a specific, reliable and sensitive measurement of specific anaerobic performance in elite female Aerobic Gymnastics, presenting great potential to be largely applied in training settings.

## Introduction

Aerobic Gymnastics (AG) has become increasingly popular over the past two decades, with currently more than 70 countries across five continents practicing this modality. AG athletes continuously perform high-intensity and complex movements following music patterns, which require high levels of fitness, strength and flexibility. According to the International Federation of Gymnastics, gymnasts have 90 seconds to execute their movement′s sequence (known as "routine") comprising seven basic elements on a 10 x 10 m area. Individuals are graded on elements difficulty and choreography, contributing to the gymnast’s performance score [1].

In spite of a growth in gymnastic modalities, few studies have investigated performance-predictive parameters in gymnastics [2–7]. Anthropometric characteristics, flexibility, aerobic capacity, and anaerobic power seem to be important factors for the execution of both rhythmic and artistic gymnastics routines [2]. Accordantly, some authors have reported that a performance of increased technical difficulty requires greater anaerobic power in artistic gymnastics [5,8,9]. In fact, anaerobic metabolism comprises around 50% of energy contribution in rhythmic gymnastics [6]. Despite this, the anaerobic demand in AG is as yet unexplored. Considering high-intensity movements and total routine time, we hypostatized that anaerobic metabolism is determinant for AG performance. In fact, preliminary data of our group and others have demonstrated high blood (Kikushi—unpublished data) and plasma (Borelli—unpublished data) lactate levels (>10 mmol/L) after an AG routine.

It is nearly impossible to reflect the specific muscular involvement and movement patters of a particular sport in laboratory′s physical tests [10]. This is particularly true for AG due to the specific and complex movements. Conversely, specific field tests allow controlled simulation of sports-related performance with relevant applications for both applied research and real training settings. For instance, specific field tests may be used by coaches to monitor athletes throughout an annual training plan. In order to validate a specific performance test, three factors have to be considered: concurrent validity, reliability, and sensitivity [11]. To date, there are no valid specific tests for measuring performance in AG.

Therefore, the aim of the current investigation was to develop a valid, reliable and sensitive specific field test designed to evaluate anaerobic physical performance in AG athletes.

## Methods

### Design of a specific field test

We first designed the Specific Aerobic Gymnast Anaerobic Test (SAGAT; also known as “Borelli′s test”). The current SAGAT version includes gymnastic-specific elements performed in maximal repeated sprint fashion, during a total timeframe of 80–90 s. Individuals must complete the test in as short a time as possible, with time trial used as the performance measure.

**Fig 1:**
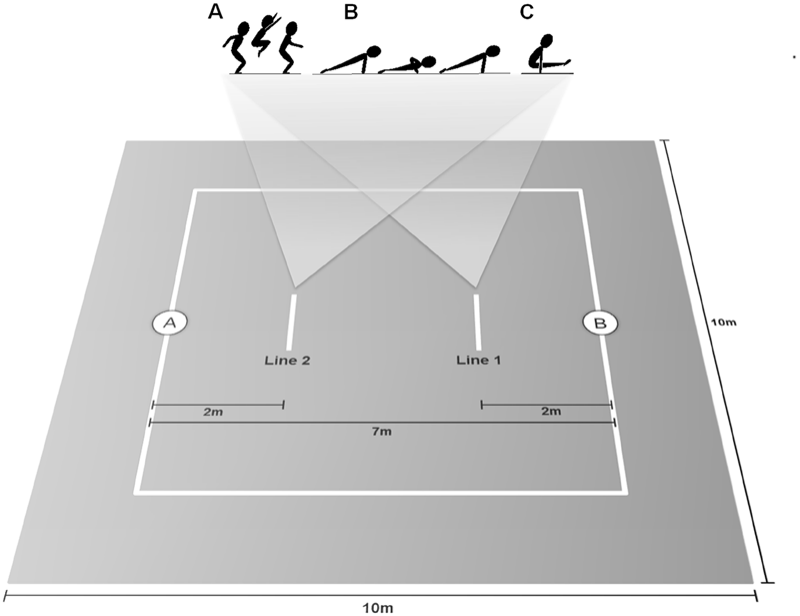
SAGAT elements and applied field illustration. SAGAT begins with the athlete standing on the “position A”. After the start command, the athlete touches their hand on the floor and runs seven meters to “position B”. The athlete then touches the floor and returns two meters towards “position A” (*i*.*e*. line 1). Following this, the athlete must then perform one “tuck jump” (A) followed by a drop in order to perform two “push-ups” (B) and one “L-support” (C). The gymnast then returns to “position B” and taps the floor, indicating that first bout is finished. Immediately after, the athlete starts the second bout by running seven meters to “position A”, and then repeats the same routine as described above until a total of 6 bouts are completed. After the completion of the first set, the athlete recovers for two minutes and repeats the entire 6-bout set.

The SAGAT was designed to focus on anaerobic metabolism performance and includes three elements of AG Punctuation’s Code: tuck jumps (Fig 1A), push-ups (Fig 1B) and L-Supports (Fig 1C). The test is composed of 2 sets with 6 consecutive bouts each. Since SAGAT was designed to focus on anaerobic metabolism performance, a 2-min recovery period is given between the sets. Each bout comprises of the execution of short and fast displacements combined with the specific elements of gymnastics as mentioned above. As illustrated in Fig 1, the bout begins with the athlete standing on the “position A” of a 10x10m stage. After the start command, the athlete touches their hand on the floor and runs seven meters to “position B”. The athlete then touches the floor and returns two meters towards “position A” (*i*.*e*. line 1). Following this, the athlete must then perform one “tuck jump” followed by a drop in order to perform two “push-ups” and one “L-support”. The gymnast then returns to “position B” and taps the floor, indicating that first bout is finished. Immediately after, the athlete starts the second bout by running seven meters to “position A”, and then repeats the same routine as described above until a total of 6 bouts are completed. The bouts must be completed as fast as possible and the time to complete the 6 bouts is recorded; all 6 bouts comprise one set. After the completion of the first set, the athlete recovers for two minutes and repeats the entire 6-bout set. Performance is evaluated by summing the time to complete both sets.

S1 Video shows the application of one 6-bout set.

### Participants and study design

In order to validate the SAGAT, this study was comprised of three independent sub-studies that evaluated the concurrent validity (Study I, n = 8), the reliability (Study II, n = 10) and the sensitivity (Study III, n = 30) of the test. A total of 42 female athletes were recruited to the study. All athletes who participated in Study A also took part in study B. Eight athletes who took part in Study C also took park in study A and B. All athletes were engaged in high-level AG competitions for at least four years prior to data collection, and were taking part in national and/or international competitions at the time of data collection.

Participant’s characteristics are presented in Table 1.

**Table 1:** Characteristics (mean ± standard deviation) of the participants.

Variable	*Study 1*: *concurrent validity*	*Study 2*: *test-retest reliability*	*Study 3*: *sensitivity*
Number of athletes (n)	8	10	30
Age (y)	18.2 ± 2.4	17.7 ± 2.4	17.3 ± 1.9
Body mass (kg)	57.1 ± 7.9	56.7 ± 7.0	54.9 ± 6.7
Height (m)	1.62 ± 0.05	1.61 ± 0.05	1.60 ± 0.06

All procedures were approved by the ethics committee (CEP) from School of Physical Education and Sport of University of Sao Paulo (CEP n°: 403.255) and by the CONEP (*National Research Ethics Committee*) (CONEP n°: 17510613.9.0000.5391), Brazil. After being fully explained about the risks and benefits involved with participation, participants signed an informed consent form. Written informed consent was obtained from the next of kin, caretakers, or guardians on behalf of the minors/children enrolled in this study. The athletes were not taking any pharmacological treatments and did not have any medical issue that would preclude them to participate in the study.

### Study I: Concurrent validity

**Fig 2:**
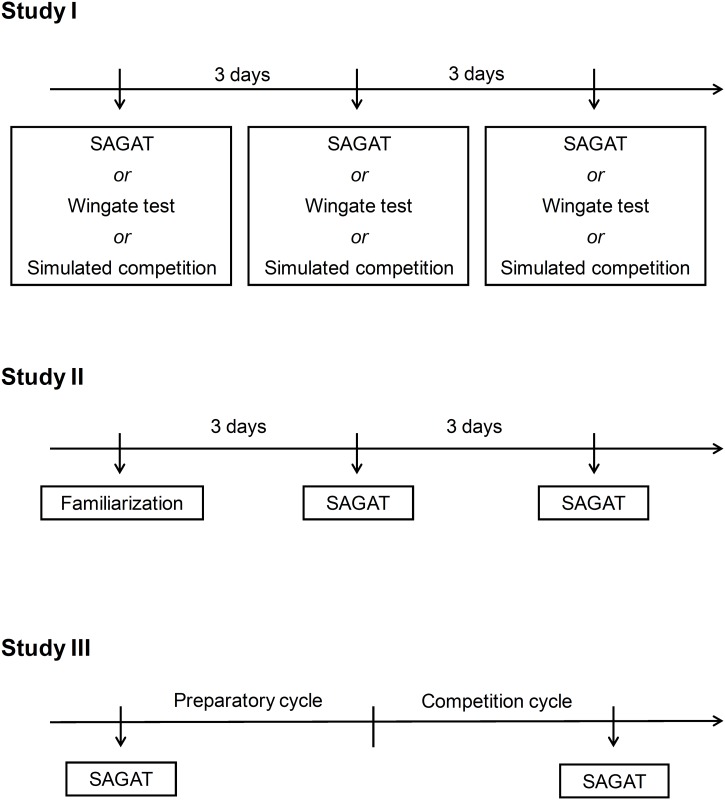
Sub-studies design: Study I—Concurrent validity, Study II—Test-retest reliability and Study III—Sensitivity.

This first study aimed to evaluate whether the SAGAT performance correlates with a previously validated anaerobic power test (*i*.*e*. the Wingate test). Additionally, metabolic demand, as assessed by plasma lactate responses, was compared between SAGAT, the Wingate test and a simulated competition. The data were collected four weeks before the national championship, a timeframe in which the athletes were likely close to their peak performance. The athletes performed three tests; *1)* the SAGAT; *2)* an upper- and a lower-body Wingate Test, and; *3)* a simulated competition. All tests were conducted on different days, three days apart, with the sequence of the tests being randomized (Fig 2).

The upper- and lower-body Wingate tests [13] were performed using a specifically designed ergometer (Cefise, Brazil). Each test was preceded by a standardised 5-minute warm-up, followed by a 5-minute resting period. Afterwards, the athletes cranked at the highest possible speed for 30 seconds against a resistance of 0.055 kp·kg^-1^ and 0.075 kp·kg^-1^ body weight for upper- and lower-body Wingate tests, respectively. A set of 24 sensors measured wheel velocity, with power output being calculated automatically every second by computer software (Ergometric 6.0, Cefise, Brazil). The highest external power output produced throughout the test was used to represent peak power, whereas the average of the power generated over the 30 seconds corresponded to mean power.

In order to simulate the atmosphere of real competitions, each athlete performed a specific competitive routine (*i*.*e*., official competition choreography) in the presence of judges, coaches, and an audience. The routines lasted 90 seconds in duration.

Capillary blood samples were collected for plasma lactate determination before and 3 minutes after each testing session (SAGAT, upper-body Wingate Test, lower-body Wingate Test, and simulated competition). Samples were stored in an ice-cold 2% NaF solution and kept on ice until centrifugation. Plasma was then separated from erythrocytes by centrifuging for 5 min at 2000 g and subsequently submitted to lactate determination using the enzymatic-colorimetric method as supplied by a commercially available kit (*Lactato*, *BioTécnica*, *MG*, *Brazil*). All samples were analysed in triplicate.

### Study II: Test-retest reliability

After being familiarised with the SAGAT, the athletes were submitted to two SAGAT tests on different days. The three sessions (*i*.*e*., familiarization, test, and retest) were separated by three days (Fig 2). All tests were applied between 2 and 3 p.m., before their regular training session. All athletes received strong verbal encouragement from their coaches and colleagues throughout the execution of the tests.

### Study III: Sensitivity

In order to examine whether the SAGAT is sensitive to detect changes in performance due to specific training, the athletes were tested on two different occasions: prior to the preparatory phase and during the competitive phase (Fig 2). These two phases of training were chosen because the athletes are expected to be below their best technical and physical performance during the preparatory phase (beginning of the season), whilst they are expected to approach their peak performance during the competitive phase.

### Statistical analysis

Pearson correlations were applied between the performance scores in SAGAT and Wingate tests. Additionally, a one-way ANOVA was performed to test differences between delta lactate responses (post exercise—rest) in each of the tests in Study I. Tukey’s multiple comparisons were used whenever a significant F value was found.

The SAGAT performance in the Study II was tested for systematic errors using a paired *t* test between the time to complete SAGAT at both test and retest [15]. Moreover, Cohen’s *d* (retest minus test divided by the standard deviation pooled) was used to determine the effect size [16]. In order to calculate the intraclass coefficient (ICC), a repeated-measures ANOVA was performed in test-retest trials and the output was applied in the following two-way random model equation: ICC = (SMS—EMS) / [SMS + (k -1) × EMS + k × (TMS—EMS) / n], where SMS = Subjects mean square, EMS = Errors mean square, TMS = trials mean square and k = numbers of trials. The minimal detectable change 95% (MDC_95_) were estimated as following: MDC_95_ = 1.96 × SEM × √2, where SEM = SD √1-ICC (for details, please see [11]).

The impact of specific AG training on SAGAT performance (Study III) was tested by means of a paired t test. Additionally, we assessed whether the change in performance over time is higher or not than the MDC_95_ found previous in the Study II.

Data are expressed as means ± standard deviations or confidence interval (CI) when appropriated. The significance level was previously set at p < 0.05.

## Results

### Study I: Concurrent validity

Descriptive data for the lower-body Wingate test, upper-body Wingate test and SAGAT performances are shown in Table 2.

**Table 2:** Descriptive performance in lower-body Wingate test, upper-body Wingate test and SAGAT (mean ± standard deviation).

**Test**	Peak power (W)	Mean power (W)	Time trial (s)
Lower-body Wingate test	499.0 ± 94.3	380.8 ± 75.6	-
Upper-body Wingate test	285.9 ± 87.6	206.0 ± 54.2	-
SAGAT	-	-	80.7 ± 3.1 s

**Fig 3:**
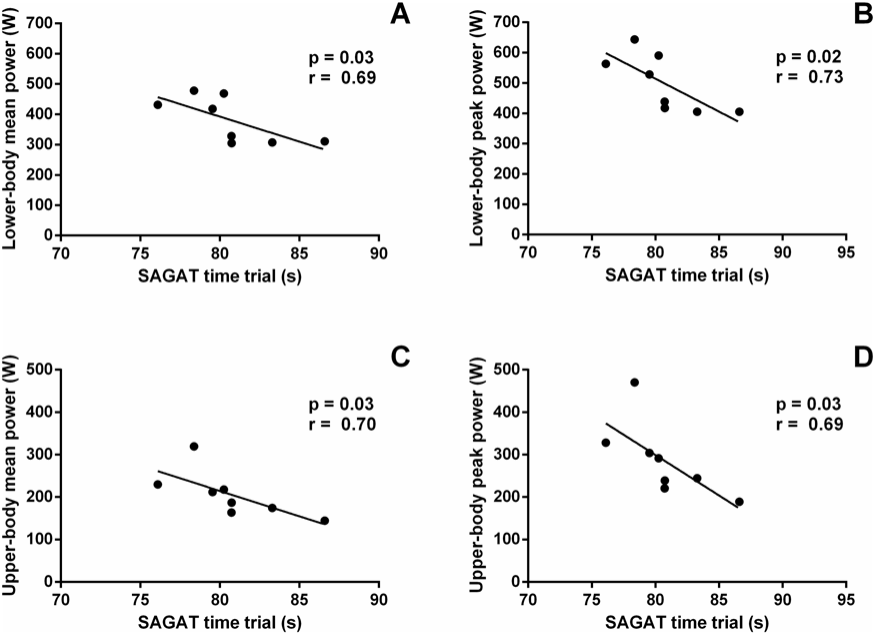
Correlations between SAGAT and Wingate test performance. Denotes significantly correlations between SAGAT time trial and lower-body Wingate test mean (Panel A; p = 0.03, r = -0.69, CI: -0.94 to 0.03) or peak (Panel B; p = 0.02, r = -0.72, CI: -0.95 to -0.04) power; and between SAGAT time trial and upper-body Wingate test mean (Panel C; p = 0.03, r = -0.67, CI: -0.94 to 0.02) or peak (Panel D; p = 0.03, r = -0.69, CI: -0.94 to 0.03) power.

A positive correlation was found between the mean and peak power obtained in the lower-body Wingate test and the SAGAT time trial (Fig 3A and 3B; p = 0.03, r = -0.69, CI: -0.94 to 0.03 and p = 0.02, r = -0.72, CI: -0.95 to -0.04, respectively). Similarly, a positive correlation was found between the mean and peak power obtained in the upper-body Wingate test and the SAGAT time trial (Fig 3C and 3D; p = 0.03, r = -0.67, CI: -0.94 to 0.02 and p = 0.03, r = -0.69, CI: -0.94 to 0.03, respectively).

**Fig 4:**
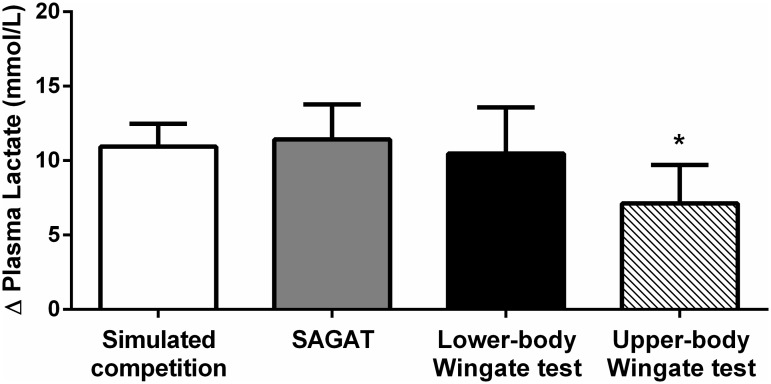
Plasma lactate response to simulated competition, SAGAT and Wingate test. Denotes no significantly differences between the simulated competition, SAGAT and lower-body Wingate test (p > 0.05 for all). *indicates significantly difference in upper-body Wingate test when compared to the simulated competition (p = 0.007), SAGAT (p = 0.002) and lower-body Wingate test (p = 0.021). Data are expressed as means ± standard deviations (error bars).

As shown in Fig 4, plasma lactate increased in response to all tests. However, the increase in plasma lactate following the upper-body Wingate Test was smaller than the increase in lactate following the lower-body Wingate Test, SAGAT and simulated competition. No significant differences were observed between these latter tests.

### Study II: Test-retest reliability

**Fig 5:**
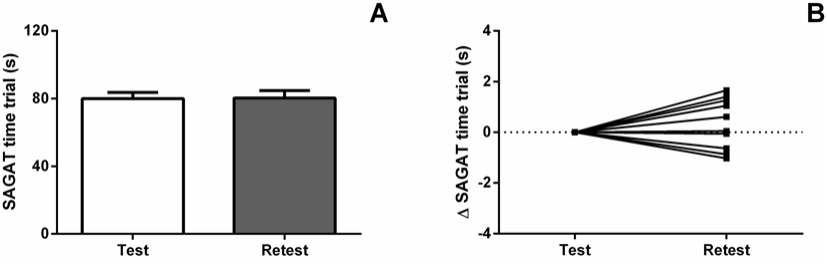
SAGAT reproducibility. Denotes no significantly differences between test and retest time trial. Data expressed as mean ± standard deviation (error bars) (A) and individual data variation (B).

ICC revealed a strong reliability index between test-retest trials (ICC = 0.97, CI: 0.89 to 0.99). As the effect of measurement error is considered minimal when ICC increases above 0.80 [17], the lower bound of the confidence interval (0.89) suggests that the current study has adequate sample size. No significant differences were found between the time to complete the SAGAT between trials (p = 0.84; Fig 5). The average variation between test and retest trials were 0.36 s, or 0.44%. Moreover, a small effect size (Cohen’s *d* = 0.09) was observed and the MDC_95_ resulting from the ICC was 0.12 s.

### Study III: Sensitivity

**Fig 6:**
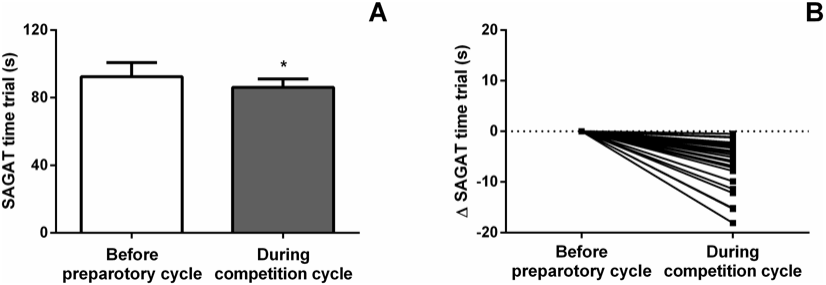
SAGAT time trial in two points of annual training plan: before preparatory cycle and during competition cycle. *Denotes significantly differences between the period before preparatory cycle and the period during competition cycle (p < 0.001). Data expressed as mean ± standard deviation (error bars) (A) and individual data variation (B).

Illustrated in Fig 6, the time to complete the SAGAT was significantly lower during the competition cycle (86.1 ± 5.0 s) when compared to the period before the preparatory cycle (92.5 ± 8.3 s; p < 0.001), showing an improvement in SAGAT performance after a specific AG training period. Importantly, the average delta change observed in the current study (6.4 s) is extremely superior to the MDC_95_ (*i*.*e*. the magnitude of change necessary to exceed the measurement error of two repeated measures at the 95% CI) found previous in the Study II.

## Discussion

In this study, we developed and validated a field test designed to evaluate specific anaerobic performance in AG. The SAGAT was created based on the technical and physiological demands of AG. Our data show that SAGAT is valid, reproducible, free of systematic errors, and sensitive to detect changes in specific performance. Since the SAGAT is easy to run and does not require expensive equipment, we believe that it may assist coaches and trainers to develop and monitor their athletes throughout training seasons.

AG is a high-intensity intermittent sport, in which competitions last 90 seconds. Thus, it is conceivable that performance would be highly dependent on the energy transferred via anaerobic metabolism. Confirming this hypothesis, we found elevated plasma lactate levels following a simulated AG competition, which was comparable to the lactate values found after the lower-body Wingate test and superior (2 to 3 fold) to those observed in rhythmic (~4.0 mmol/l) and artistic gymnastics (~5.5 mmol/l) [6,12]. In view of this high anaerobic demand, the SAGAT was designed to elicit a large activation of the glycolytic pathways, thereby resulting in lactate responses comparable to those observed after competitions. In that respect, our data have confirmed that the SAGAT is metabolically similar to a simulated AG competition.

SAGAT was designed to reflect the total duration and intensity of typical AG competitions. The similar lactate responses between the SAGAT and the simulated AG competition, in addition to the positive correlations between SAGAT and performance in the upper- and lower-body Wingate tests, suggest that SAGAT is effective to measure anaerobic metabolism in AG athletes. Although the upper-body Wingate Test yielded a smaller lactate response, it must be noted that this is probably a result of a smaller muscle mass that is active during the upper-body test as compared to lower-body or whole-body exercises [13].

Reliability and sensitivity are two other important characteristics of a valid field test [11,14]. Reliability refers to the normal variation of a test and it is generally accepted that a reproducible test should exhibit 1) no differences between two similar measures (*e*.*g*., test-retest measures) [11] and, 2) a coefficient of variation lower than 5% [11]. In this context, the same group of athletes performing the SAGAT in similar conditions and only three days apart were able to perform similarly, with no differences, low effect size and a low coefficient of variation between two trials. This indicates that the SAGAT provides a stable and reliable specific performance test free of systematic-errors index.

A sensitive test must be able to detect small changes in performance after a period of training. In the case of a specific test, a specific training period should reflect improvements in specific performance. In line with this, an improvement in SAGAT performance after ~8 months of AG training demonstrated that the SAGAT also has desirable sensitivity.

Despite being valid, specific, easy to be applied and cost-effective, the SAGAT has some limitations. In order to increase the specificity of test, SAGAT was designed to include elements that are generally observed in AG competitions (*i*.*e*. Tuck Jump, Push-up, and L-Support). However, the technical execution of these elements must be compatible with the athlete’s best execution in order for the test to be considered valid. Thus, the SAGAT needs to be applied by an experienced coach and the technical elements must be subjectively evaluated throughout the test. Whenever a given athlete does not complete the skill elements correctly, the test should be cancelled (for details see the S1 Video). Moreover, the present study also has some limitations. First, our group of athletes included only female elite gymnasts. Thus, the validity of the SAGAT in different populations, especially in non-elite and less skilled gymnasts, is yet to be determined. Secondly, this is the first study, to our knowledge, to report an indicator of the energy demand in AG. Although our lactate results are in accordance with what would be expected after a whole-body high-intensity exercise lasting 90 s, a full investigation of the physiological demands including estimates of aerobic and anaerobic metabolism is still necessary in both AG competition and SAGAT.

In conclusion, we demonstrated that SAGAT is a specific, reliable and sensitive measurement of anaerobic performance in elite female AG gymnasts, with great potential to be largely applied in training settings, assisting coaches and trainers to monitor appropriate training programs in the sport.

## Supporting Information

S1 Video One 6-bout set of SAGAT test application. (MP4)
